# Supplementary material for: Retinal Microvascular Abnormalities Predict Clinical Outcomes in Patients with Heart Failure
Source: Diagnostics (Basel). 2022 Aug 27;12(9):2078. doi: 10.3390/diagnostics12092078 (PMC9497585; doi:10.3390/diagnostics12092078)
Supplement: Supplementary file 1 [file diagnostics-12-02078-s001.zip › diagnostics-1865461-supplementary.pdf]

Supplemental Table S1. Demographic and baseline characteristics of HF patients with CRVE ≥ 283 μm versus HF patients without endpoints

variables	HF patients with CRVE ≥ 283 μm (n=9)	HF patients with CRVE < 283 (n=46)	P value
Age (years)	57.22 ± 14.11	67.26 ± 11.91	0.029
Male (%)	7 (77.8)	32 (69.6)	0.620
Hospital stay (days)	7.67 ± 3.24	9.52 ± 3.97	0.770
Systolic BP (mmHg)	149.44 ± 36.16	137.57 ± 24.43	0.225
Diastolic BP (mmHg)	92.22 ± 14.34	86.13 ± 17.17	0.323
Heart rate (b.p.m.)	83.00 ± 10.35	86.04 ± 22.41	0.693
Etiology of HF			
Ischemic (%)	4 (44.4)	19 (41.3)	0.593
Dilated (%)	2 (22.2)	5 (10.9)	
Other (%)	3 (33.3)	22 (47.8)	
NYHA class			
I (%)	0 (0)	0 (0)	0.758
II (%)	3 (33.3)	10 (21.7)	
III (%)	5 (55.6)	29 (63.0)	
IV (%)	1 (11.1)	7 (15.2)	
**Medical history**			
Hypertension (%)	6 (66.7)	31 (67.4)	0.966
Diabetes mellitus (%)	5 (55.6)	21 (45.7)	0.858
Myocardial farction (%)	2 (22.2)	11 (23.9)	0.913
Revascularization (%)	3 (33.3)	12 (26.1)	0.655
Atrial fibrillation (%)	6 (66.7)	17 (37.0)	0.199
Dyslipidemia	4 (44.4)	12 (26.1)	0.267
Stroke (%)	1 (11.1)	13 (28.3)	0.508
Smoker (%)	3 (33.3)	14 (30.4)	0.863
**Echocardiographic indicators**			
LVEF (%)	40.0 (26.0 - 56.0)	40.0 (30.0 - 57.5)	0.767
LAD (mm)	47.0 (43.0 - 63.0)	49.7 (44.9 - 55.8)	0.915
LVESD (mm)	53.6 (30.0 - 62.0)	44.8 (31.0 - 53.2)	0.612
LVEDD (mm)	61.7 (46.0 - 69.3)	57.9 (49.3 - 61.9)	0.456
Septal wall thickness (mm)	9.7 (9.1 - 14.4)	9.6 (8.1 - 11.6)	0.260
Posterior wall thickness (mm)	9.5 (7.2 - 9.9)	9.5 (8.5 - 11.6)	0.991
Septal E/e'	20.9 (13.0 - 26.9)	20.7 (14.5 - 28.9)	0.692
Pulmonary arterial hypertension (%)	3 (37.5)	13 (29.5)	0.654
**Laboratory**			
Triglycerides (mM)	1.34 ± 0.66	1.27 ± 0.92	0.840
Total cholesterol (mM)	4.76 ± 0.96	4.22 ± 1.32	0.273
LDL (mM)	3.29 ± 0.69	2.67 ± 1.04	0.115
HDL (mM)	0.95 ± 0.27	1.07 ± 0.32	0.319
Fasting glucose (mM)	6.17 (5.13 – 6.81)	6.18 (4.89 – 8.44)	0.678

Creatinine (μM)	135.09 ± 66.93	97.45 ± 41.74	0.030
eGFR (mL/min/BSA)	62.49 (28.83 - 83.98)	78.61 (57.04 - 94.03)	0.439
NT-proBNP (ng/L)	3621.00 (1570.00 – 24615.00)	2680.00 (1471.50 - 7665.50)	0.939
Troponin T (ng/ml)	0.02 (0.01 - 0.26)	0.01 (0.00 - 0.04)	0.084
Haemoglobin (g/L)	127.67 ± 69.58	133.26 ± 19.48	0.516
**Medication**			
ACEI/ARB/ARNI (%)	6 (66.7)	37 (80.4)	0.360
Beta-blocker (%)	6 (66.6)	37 (80.4)	0.360
Loop diuretic (%)	16 (72.7)	21 (63.6)	0.481
Stain	4 (44.4)	32 (69.9)	0.147
Mineralocorticoid receptor antagonist (%)	5 (55.6)	31 (67.4)	0.495
Calciμm channel blocker (%)	2 (22.2)	12 (26.1)	0.808
Oral anticoagulation (%)	4 (44.4)	15 (32.6)	0.495
**Retinal vessel calibers**			
CRAE (μm)	165.94 (123.14 - 174.68)	158.76 (147.54 - 168.69)	0.982
CRVE (μm)	293.33 (288.58 - 303.84)	253.58 (237.29 - 266.81)	<0.001
AVR	0.55 (0.42 - 0.59)	0.63 (0.59 - 0.68)	0.001
